# Acid-Sensing Ion Channels: Expression and Function in Resident and Infiltrating Immune Cells in the Central Nervous System

**DOI:** 10.3389/fncel.2021.738043

**Published:** 2021-09-17

**Authors:** Victoria S. Foster, Lachlan D. Rash, Glenn F. King, Michelle M. Rank

**Affiliations:** ^1^Institute for Molecular Bioscience, The University of Queensland, St Lucia, QLD, Australia; ^2^School of Biomedical Sciences, The University of Queensland, St Lucia, QLD, Australia; ^3^Australian Research Council Centre of Excellence for Innovations in Peptide and Protein Science, The University of Queensland, St Lucia, QLD, Australia; ^4^Anatomy and Physiology, Medicine, Dentistry and Health Sciences, The University of Melbourne, Melbourne, VIC, Australia

**Keywords:** acid-sensing ion channel (ASIC), central nervous system, immune cell, acidosis, neuropathology, neuroimmunology, ion channel

## Abstract

Peripheral and central immune cells are critical for fighting disease, but they can also play a pivotal role in the onset and/or progression of a variety of neurological conditions that affect the central nervous system (CNS). Tissue acidosis is often present in CNS pathologies such as multiple sclerosis, epileptic seizures, and depression, and local pH is also reduced during periods of ischemia following stroke, traumatic brain injury, and spinal cord injury. These pathological increases in extracellular acidity can activate a class of proton-gated channels known as acid-sensing ion channels (ASICs). ASICs have been primarily studied due to their ubiquitous expression throughout the nervous system, but it is less well recognized that they are also found in various types of immune cells. In this review, we explore what is currently known about the expression of ASICs in both peripheral and CNS-resident immune cells, and how channel activation during pathological tissue acidosis may lead to altered immune cell function that in turn modulates inflammatory pathology in the CNS. We identify gaps in the literature where ASICs and immune cell function has not been characterized, such as neurotrauma. Knowledge of the contribution of ASICs to immune cell function in neuropathology will be critical for determining whether the therapeutic benefits of ASIC inhibition might be due in part to an effect on immune cells.

## Acid-Sensing Ion Channels

**FIGURE 1:**
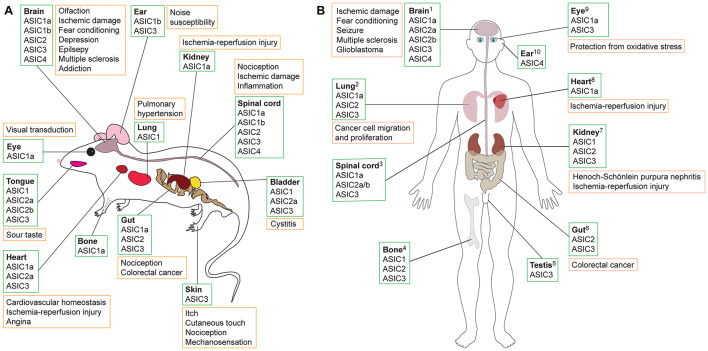
Schematic showing tissues in which ASICs are expressed (green boxes) and their putative role in various pathologies (orange boxes) in **(A)** rodents and **(B**) humans. Data summarized here are drawn from Supplementary Table 1, with expression sites and pathology based on studies that used antibody staining, transcriptomic data, proteomics and/or functional methods. Additional information is available in the Human Protein Atlas (www.proteinatlas.org). References supporting the putative roles of ASICs in human pathologies are as follows: ^1^**Brain:** ASIC1a detected using RT-PCR and Western blot; acidosis-mediated damage to cultured human brain neurons rescued by ASIC1a inhibition with PcTx1 (Li et al., 2010). Significant association between ASIC1a SNPs, amygdala volume, and panic disorder in humans (Smoller et al., 2014). Elevated levels of ASIC1a (Yang et al., 2016) and ASIC3 (Cao et al., 2016) in brains of patients with temporal lobe epilepsy shown using immunostaining and Western blot. ASIC expression in CNS; treatment with amiloride alleviated patient symptoms of MS (Arun et al., 2013). ASIC1 and ASIC3 found in glioblastoma stem cell lines using RT-PCR and Western blot; microarray data revealed that ASIC expression is associated with improved survival in glioma patients (Tian et al., 2017). ^2^**Lung:** ASICs expressed in human lung cancer cell line A549 determined using RT-PCR, immunofluorescence, and Western blot. Proliferation and migration promoted by overexpression of ASIC1a and inhibited by PcTx1 (Wu et al., 2017). ^3^**Spinal cord:** ASICs in human spinal cord, stained with ASIC1 antibody (Arun et al., 2013). ^4^**Bone:** ASIC expression in human skeleton shown using RT-PCR and antibody staining performed on human chondrocytes (Jahr et al., 2005). ^5^**Testis:** ASIC3 in human testis based on Northern blot (Ishibashi and Marumo, 1998). ^6^**Gut:** ASIC2 measured using RT-PCR and immunostaining in human colorectal cancer cells, with ASIC2 promoting cell invasion and proliferation in xenografts (worsened via overexpression and impeded with ASIC2 knockout) (Zhou et al., 2017). ^7^**Kidney:** ASICs found in human proximal tubular cell line using RT-PCR and Western blotting; apoptosis of cells due to ischemia-reperfusion injury reduced by ASIC1a inhibition using PcTx1 (Song et al., 2019). ASIC1, ASIC2 and ASIC3 protein expression in patients with Henoch-Schönlein purpura nephritis; ASIC blockade with amiloride reduced expression of damage marker proteins (Yuan et al., 2010). ^8^**Heart:** Transcriptomics revealed presence of ASIC1a in human induced pluripotent stem cell-derived cardiomyocytes (hiPSC-CMs); ASIC1a inhibition with Hi1a or PcTx1 improved viability of hiPSC-CMs under conditions of hypoxia and acidosis, whereas viability was further reduced in the presence of the ASIC1a activator MitTx (Redd et al., 2021). ^9^**Eye:** ASIC1a detected in retinal pigment epithelial cells using RT-PCR and Western blotting; PcTx1 protects cells from oxidative stress (Tan et al., 2013). ASIC3 mRNA detected using RT-PCR on human retina samples (Maubaret et al., 2002). ^10^**Ear:** Presence of ASIC4 shown using RT-PCR and Northern blot (Gründer et al., 2000).

Acid-sensing ion channels (ASICs) are proton-gated ion channels that are permeable to Na^+^ (Waldmann et al., 1997), and they constitute a subfamily of the epithelial sodium channel/degenerin (ENaC/Deg) superfamily (Kellenberger and Schild, 2015). ENaCs facilitate Na^+^ reabsorption in the kidney, and they regulate the volume in the fluid/cilia interface in both the lung and colonic epithelial cells (Garty and Palmer, 1997; Enuka et al., 2012; Hanukoglu and Hanukoglu, 2016). ASICs have ∼30% sequence identity to ENaCs, and both groups are inhibited by the diuretic drug amiloride (Paukert et al., 2004). However, in contrast to ENaCs, ASICs appear to have evolved earlier, possibly first appearing in deuterostomes ∼600 million years ago (Lynagh et al., 2018). Humans possess four ASIC-coding genes (*ASIC1*–*ASIC4*), three of which (*ASIC1, ASIC2* and *ASIC3)* are alternatively spliced, to produce six main subunits with differing properties: ASIC1a, ASIC1b, ASIC2a, ASIC2b, ASIC3a, and ASIC4 (Table 1; Wemmie et al., 2013). ASIC3a is the major splice isoform of *ASIC3*, with little known about the function of ASIC3b and ASIC3c (Delaunay et al., 2012). ASICs form homotrimeric or heterotrimeric channels (Jasti et al., 2007) that have different pH thresholds for channel activation (see Table 1), different physiological and pathological roles, and different tissue distribution (Figure 1; Waldmann et al., 1997, 1999; Chen et al., 1998). ASIC1a homomers, ASIC1a/2b heteromers (but not ASIC1a/2a), and human ASIC1b mediate the influx of Ca^2+^ in addition to Na^+^, although they are all more permeable to Na^+^ (Waldmann et al., 1997; Olena et al., 2004; Hoagland et al., 2010; Sherwood et al., 2011).

**TABLE 1:** ASIC subtypes and their pH sensitivity.

**Gene**	**Subtype**	**Alternative names**	**pH sensitivity (pH_50_)^1^**
*ASIC1*	ASIC1a	ASIC, ASICα, BNaC2(α)	5.8–6.8
	ASIC1b	ASICβ, BNaC2(β)	6.1–6.2
*ASIC2*	ASIC2a	BNaC1(α), MDEG, BNC(1a)	4.5–4.9
	ASIC2b	BNaC1(β), MDEG2	Does not form pH-sensitive homomers; associates with other ASICs to form pH-sensitive channels
*ASIC3*	ASIC3	DRASIC, TNaC1, SLNAC1	6.4–6.6
*ASIC4*	ASIC4	BNAC4, ACCN4, SPASIC	Does not form pH-sensitive homomeric channels

*^1^The pH sensitivities of the homomeric ASIC channels are from Wemmie et al. (2013).*

ASICs are expressed in a range of tissues and have been associated with diverse pathologies, including diabetes, stroke, myocardial infarction, and epilepsy (see Figure 1; Xiong et al., 2004; Lv et al., 2011; Radu et al., 2014; Chassagnon et al., 2017; Redd et al., 2021). Most ASIC subtypes are present in the peripheral nervous system (PNS). However, ASIC1a is expressed at high levels and is the dominant subtype in both the human and rodent central nervous system (CNS) (Sluka et al., 2003; Wemmie et al., 2003; Baron et al., 2008), and it has been implicated in a variety of CNS disorders such as neurodegenerative diseases, depression, epilepsy, and ischemia-induced injury of the brain and spinal cord (Xiong et al., 2004; Friese et al., 2007; Wong et al., 2008; Ziemann et al., 2008; Coryell et al., 2009; Hu et al., 2011; Lv et al., 2011; Radu et al., 2014; Koehn et al., 2016; Chassagnon et al., 2017).

## Inflammation

There is a growing body of literature suggesting that ASICs may contribute to immune cell function and neuroinflammation during CNS pathology. Inflammation is a complex biological response to an insult, which may be pathogenic or self-derived (i.e., induced by trauma, ischemia, or autoimmune processes). It is driven by the innate immune system, dependent on immune cell infiltration, and results in the release of plethora of inflammatory mediators including histamines, bradykinins, arachidonic acid, leukotrienes, prostaglandins, cytokines and chemokines. Plasma leakage from capillary beds causes swelling, alongside extravasation of granulocytes into the tissue which is facilitated by P-selectin and platelet endothelial cell adhesion molecule 1 (PECAM-1) (Woodfin et al., 2007). Chronic inflammation causes long term alterations to cell populations, such as an increase in the number of white blood cells such as lymphocytes. This infiltration and persistent involvement of immune cells results in both healing alongside further damage. Neuroinflammation is an inflammatory response within the CNS, leading to immune cell infiltration (of CNS or peripheral origin) and increased levels of cytokines, chemokines, and reactive oxygen species (ROS). It is worth noting that inflammation can be “sterile,” occurring without a pathogenic external source [e.g., pathogen associated molecular patterns (PAMPs)] and instead resulting from damage-associated molecular patterns (DAMPs) triggered by trauma, ischemia or other environmental factors (e.g., ultraviolet radiation) (Rock et al., 2010; Feldman et al., 2015).

Neuroinflammation has been implicated in degenerative and traumatic conditions and even mental health disorders such as depression and anxiety. Despite robust intrinsic CNS barriers, resident peripheral immune cells frequently cross into the CNS parenchyma when the blood-brain barrier (BBB), blood-spinal cord barrier (BSCB), or blood-cerebrospinal fluid barrier (BCSFB) become porous. The BBB, BSCB, and BCSFB are often disrupted and left partially open for weeks after a physical injury such as spinal cord injury (SCI) and traumatic brain injury (TBI), or after ischemic insults such as stroke (Sinescu et al., 2010; Chodobski et al., 2011; Suzuki et al., 2016). Degenerative disorders such as Alzheimer’s disease (AD), Parkinson’s disease (PD), and multiple sclerosis (MS) also cause pervasive disruption to CNS barriers that results in CNS invasion by peripheral immune cells (Ffrench-Constant, 1994; Town et al., 2005; Polman et al., 2006). Breakdown of the BBB is also thought to contribute to the etiology of epilepsy (Marchi et al., 2012).

Neutrophils, macrophages, T cells, and dendritic cells (DCs) have each been shown to occupy the CNS after an insult. Neutrophil chemoattractants such as PGF2α, complement component C5a, and interleukin-8 (CXCL-8) are produced by ischemic tissues, suggesting that these leukocytes do not just passively filter through the disrupted BBB/BSCB but are actively recruited to the CNS following the insult (Kilgore et al., 1994; Arnould et al., 2001; De Bondt et al., 2020). Consistent with this, neutrophil migration is altered by acidity, which is discussed further below (Rotstein et al., 1988). There is conflicting evidence as to whether neutrophils are beneficial or detrimental once they have infiltrated the CNS, which may be due to the heterogenous nature of neutrophil activation states (e.g., differential expression of proteins), but they are accepted to contribute to SCI severity (Taoka et al., 1998; Neirinckx et al., 2014; Deniset and Kubes, 2018). Infiltrating macrophages/monocytes and possibly neutrophils initiate demyelination of CNS neurons through phagocytic and inflammatory processes (Ajami et al., 2011; Yamasaki et al., 2014; Stranahan et al., 2016; De Bondt et al., 2020). T cells that cross a porous BBB contribute significantly to MS pathology by causing central demyelination through attack and degradation of the myelin sheath (Bielekova et al., 2004). During experimental autoimmune encephalomyelitis (EAE), an animal model of MS, DCs also breach the CNS and subsequently prime T cells to exacerbate the autoimmune inflammation (Karman et al., 2006; Sagar et al., 2012). In summary, recruitment of peripheral immune cells into the CNS during inflammation contributes to the wave of secondary damage that emanates from the initial site of injury.

## Tissue Acidification

**FIGURE 2:**
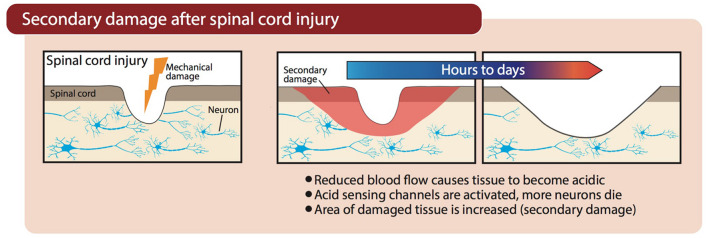
Progression of secondary damage after physical damage is inflicted on the spinal cord. Blood flow is restricted as a result of damaged blood vessels, causing tissue acidification and subsequent activation of ASICs.

Vascular damage after SCI, hemorrhagic stroke, or occlusion of cerebral arteries during an ischemic stroke, causes a marked reduction in blood flow to the injured region. Neurons and supporting glial cells in the center of the ischemic territory (i.e., where blood flow is lowest) are rapidly, and perhaps irreparably, damaged. At the periphery of this ischemic core (the penumbra), an expanding wave of secondary damage (i.e., progression of injury beyond the initial insult, as outlined in Figure 2) develops more slowly because supplementary blood flow from adjacent regions maintains perfusion above the threshold for immediate cell death (Moskowitz et al., 2010). However, in the absence of adequate blood flow, oxygen deprivation (hypoxia) in areas surrounding the primary ischemic insult forces neurons to resort to anaerobic glycolysis for their energy needs, which causes lactic acidosis and acidification of the tissue (Rehncrona, 1985). The resultant acidosis contributes to secondary damage through activation of ASICs (e.g., ASIC1a), which is discussed further below. Patients admitted with trauma often exhibit a decrease in blood pH as part of the “triad of death”—systemic hypothermia, acidosis, and coagulopathy—which contributes to mortality and is also a reminder that in trauma, acidification is not limited to the CNS or just the site of damage (Mikhail, 1999; Mitra et al., 2012).

Acidification is not solely linked to traumatic and ischemic insults. Extracellular acidosis has been reported in spinal cord tissue of EAE mice, and increased levels of tissue lactate have also been reported in human MS brain tissue, sufficient to activate ASIC1a (Bitsch et al., 1999; Friese et al., 2007). In both animal models of Huntington’s disease (HD) and in patients with HD, a significant build-up of lactic acid has been observed in the brain (Tsang et al., 2006; Josefsen et al., 2010).

Tissue acidosis and inflammation influence one another and, as shown by research within an intensive care unit, alterations in the acid/base status of patients contribute to differences in their interleukin (IL) and cytokine profile (Zampieri et al., 2014). We have highlighted thus far that both inflammation and acidity contribute to tissue damage in pathological CNS conditions, with the former moderated by immune cells and the latter by receptors such as ASICs. Immune cells themselves are also modulated by acidity, and in the following sections we present an overview of immune cells, their response to acidic conditions, and the potential role of immune-cell ASICs in neuroinflammation.

## Overview of Immune Cells

**FIGURE 3:**
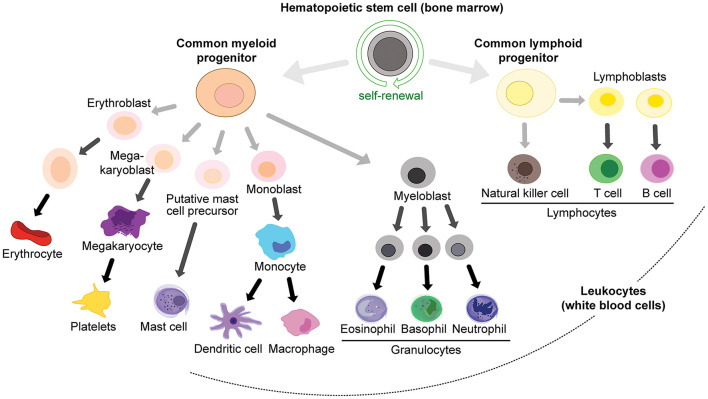
Immune cell lineages in the periphery.

White blood cell lineages (peripheral immune cells) arise from hemopoietic stem cells in the bone marrow (Figure 3), in particular the common myeloid progenitor and the common lymphoid progenitor cells. Common myeloid progenitors give rise to granulocytes (mast cells, eosinophils, basophils and neutrophils) and monocytes (which can differentiate into macrophages and DCs), while the common lymphoid progenitor produces various classes of lymphocytes: natural killer (NK) cells, T cells and B cells (Kondo et al., 2003; Figure 3). T cells are produced in the bone marrow and mature in the thymus, and they can be further subdivided into memory, cytotoxic, regulatory, and helper T cells. T cells express T cell receptors (TCRs) on their surface to recognize antigens.

**FIGURE 4:**
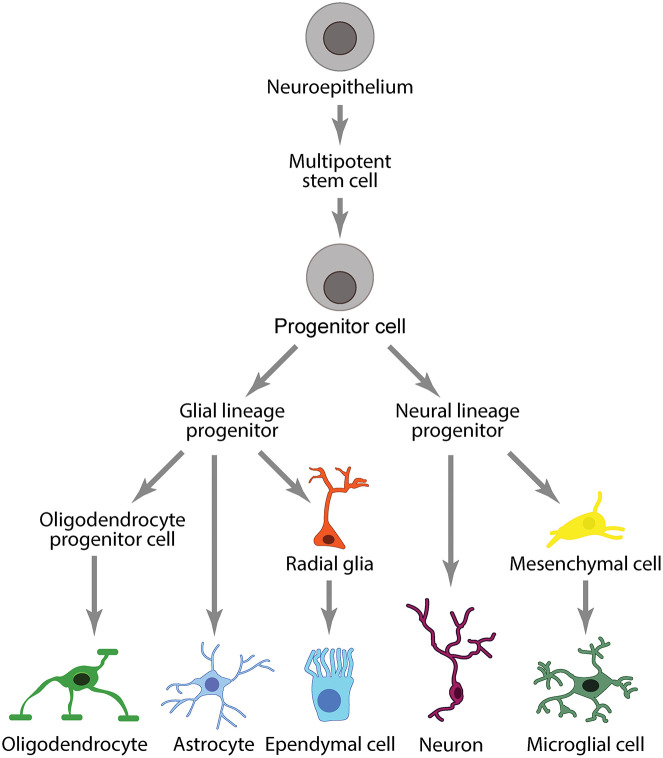
Immune cell lineages in the CNS.

Peripheral immune cells are separated from CNS immune cells by two distinct barriers that divide the central and peripheral vascular systems: the BBB/BSCB and the BCSFB. The BBB/BSCB and BCSFB allow the CNS to maintain its own regulatory environment with a distinct set of immune cells comprising the neuroglia (microglia, astrocytes, oligodendrocytes) and ependymal cells (Figure 4).

### Immune Cell Response to Tissue Acidification

Although often referred as neuronal ion channels, ASICs are expressed in numerous types of immune cells (Huang et al., 2010; Tong et al., 2011; Kong et al., 2013; Yu et al., 2015) and they are potentiated by molecules associated with neuroinflammation such as arachidonic acid, histamine, lactate, and nitric oxide (Immke and McCleskey, 2001; Cadiou et al., 2007; Smith et al., 2007; Rash, 2017; Shteinikov et al., 2017). Acidity can provoke different responses in immune cells depending on the source of the acidosis, as exemplified in Table 2 for macrophages. Therefore, whether or how inflammation is triggered by acidity depends on the source of the lowered pH. As proton-coupled monocarboxylate transporters (MCTs) shuttle lactate in a 1:1 ratio with H^+^ (Hertz and Dienel, 2005), altering lactate levels will have secondary effects on the H^+^ concentration.

**TABLE 2:** The effects of pH on inflammatory mediators in macrophages (Kellum et al., 2004).

**Acid**	**pH_*o*_^1^**	**Cells**	**LPS^2^**	**Effect**	**References**
HCl	6.5	Alveolar macrophages	+	↑TNF^3^ mRNA	Heming et al., 2001a
HCl	5.5	Alveolar macrophages	+	↑TNF mRNA ↓TNF secretion	Heming et al., 2001a
HCl	5.5	RAW 264.7 cell line^4^	+	No ΔTNF mRNA ↓TNF secretion	Heming et al., 2001b
HCl	7.0	Alveolar macrophages	+	↓TNF secretion	Bidani et al., 1998
HCl	7.0	Peritoneal macrophages	–	↑NO^5^, ↑TNF^6^, ↑NF-κB^7^	Bellocq et al., 1998
HCl	7.2	RAW 264.7 cell line	+	↑NO	Huang et al., 2002
Lactate	6.7	Peritoneal macrophages	+	↑TNF mRNA ↑TNF secretion	Jensen et al., 1990
DS^8^	6.0	Peritoneal macrophages	+	↓TNF mRNA ↓TNF secretion	Jörres et al., 1995
DS^8^	6.5	Human blood-borne macrophages	+	↓TNF mRNA ↓NF-κB	Douvdevani et al., 1995

*^1^pH_*o*_, extracellular pH.*

*^2^LPS, lipopolysaccharide.*

*^3^TNF, tumor necrosis factor.*

*^4^RAW 264.7, mouse macrophage-like cell line.*

*^5^NO, nitric oxide.*

*^6^TNF not measured directly.*

*^7^NF-κB, nuclear factor-κB.*

*^8^DS, lactate-based dialysis solution.*

Neutrophils exposed to acidic pH undergo various functional changes, including significantly impaired migration, defective chemotaxis, reduced speed of apoptosis, increased CD18 expression, and increased phagocytosis but with decreased bactericide action (Rotstein et al., 1988; Trevani et al., 1999; Cao et al., 2015). The combination of acidosis-induced decreases in migration, chemotaxis and apoptosis together with increased phagocytic activity might suggest that once neutrophils enter a region of low pH, they become trapped there with enhanced lifespan and inflammatory activity. CD18 is a cell-surface molecule expressed by neutrophils that promotes cell adhesion, a process that allows neutrophils to aid tissue repair in the periphery. Altered expression of CD18 is believed to facilitate neutrophil breach into the CNS (Serrano et al., 1996; Neri et al., 2018). The acidic blood pH after trauma (as low as 6.6) likely induces altered CD18 expression on peripheral neutrophils which then undergo facilitated extravasation into the CNS, where they accumulate in acidic regions (Mitra et al., 2012). Furthermore, in patients with SCI, neutrophils exhibit a higher “respiratory burst” in the blood (Kanyilmaz et al., 2013). The myeloperoxidase (MPO) produced by neutrophils enhances conversion of H_2_O_2_ to HOCl, and thus contributes to the secondary damage after SCI (Kubota et al., 2012). Neutrophils in the blood of SCI patients in rehabilitation have decreased ability to phagocytose *Escherichia coli* and *Staphylococcus aureus* (Campagnolo et al., 1997; Kanyilmaz et al., 2013). These data suggest that, at least for neutrophils, typical immune responses are altered in the presence of acidity and this may explain why neutrophils can have a detrimental impact in the CNS after neurotrauma.

Vermeulen et al. (2004) and Martínez et al. (2007) found that murine and human DCs mature upon exposure to acidity. Acidosis modifies the inflammatory profile of monocytes and macrophages, and is proposed to induce an inflammatory state in the latter (Riemann et al., 2016). Microglial motility decreases after exposure to acidic pH (Faff and Nolte, 2000). Rat macrophages exposed to acidic environments (pH below 7.4) upregulate nitric oxide synthase (NOS), and thus increase production of NO (Bellocq et al., 1998). Excess amounts of NO are detrimental in neurodegenerative pathologies such as HD and after striatal lesions, causing DNA damage and oxidative stress to cells (Schulz et al., 1996; Browne and Beal, 2006; Calabrese et al., 2007). These responses are discussed further below when considering the role of ASICs.

Decreased extracellular pH, therefore, can modify the function of immune cells by altering motility, impacting phagocytosis, aiding CNS invasion, and increasing the production of potentially neurodamaging agents such as NO. Although the function of neutrophils is modified by acidity, there are, to date, no studies on whether they express ASICs. Thus, the role of ASICs in acidosis-induced responses of neutrophils is currently unknown.

### Receptors and Ion Channels on Immune Cells

The key immune cell receptors involved in the innate immune response are complement and cytokine/chemokine receptors and pattern recognition receptors (PRRs) such as Toll-like receptors (TLRs). The PRRs recognize both PAMPs and also DAMPs which are produced by dying or damaged cells and trigger sterile inflammatory processes. As part of the adaptive immune response, the TCRs and antibody-binding receptors are involved in cascades that facilitate the immune cell response. Immune cells also possess ion channels that are important for their function, including the voltage-gated potassium channel Kv1.3, the calcium-activated potassium channel KCa3.1, calcium release-activated calcium channels (CRAC), and transient receptor potential (TRP) channel M7 (Cadiou et al., 2007; Chandy and Norton, 2017; Chiang et al., 2017; Nadolni and Zierler, 2018; Clemens and Lowell, 2019). The function of these ion channels in immune cells and their contribution to pathology have been reviewed (Feske et al., 2015; Vaeth and Feske, 2018) but neither of these reviews discuss the presence or role of ASICs in immune cells.

## Acid-Sensing Ion Channel Expression in Cells That Contribute to Neuropathology

### Acid-Sensing Ion Channels in Microglia

Microglia are the main immune cell type in the CNS. Although considered CNS-resident macrophages, microglia branch off earlier in development from yolk sac precursors (Ginhoux et al., 2010; Schulz et al., 2012; Kierdorf et al., 2013). In non-pathological conditions, microglia are involved in clearing tissue debris and surveying the environment (Nimmerjahn et al., 2005). During neuropathology, microglia can enter an activated state and release enzymes such as NADPH oxidase, which causes neuronal damage. Microglia can additionally modify myelination, affecting developmental myelination as well as beneficial re-myelination processes (Popovich et al., 2002; Butovsky et al., 2006). The divergent context-specific roles of microglia in CNS pathologies is an emergent field and has been recently reviewed (Norris and Kipnis, 2019; Butler et al., 2021).

It has been known for some time that the severity of disease progression in MS patients is linked to microglial activation (Heppner et al., 2005; Jack et al., 2005; Geladaris et al., 2021), and it seems that microglial phagocytosis of stressed neurons contributes to neurodegeneration in AD, PD, ischemic stroke, CNS viral infections, and aging (Butler et al., 2021). Rat microglia have been shown to express ASIC1, ASIC2a and ASIC3. In cultured primary rat microglia stimulated with lipopolysaccharide (LPS), a known immune activator, ASIC1 and ASIC2a expression increased both at the surface and intracellularly (Yu et al., 2015) and ASIC-specific inward currents could be recorded using whole-cell patch-clamp electrophysiology. Both PcTx1 and amiloride reduced the amplitude of these currents and reduced the expression of inflammatory cytokines (Yu et al., 2015). Treatment of cultured primary microglia with amiloride also decreased the extent of phagocytosis under acidic conditions (Vergo et al., 2011). A decrease in phagocytosis was also seen in microglia cultured from ASIC1^–/–^ mice, with no change upon the addition of amiloride, indicating that the effect of amiloride is ASIC1-mediated.

These data collectively show that ASICs, and ASIC1a specifically, are present in microglia and contribute to their function in acidic microenvironments. In turn, this indicates that microglial ASIC1a contributes to microglia function, affecting release of inflammatory cytokines and phagocytosis, potentially impacting the immune response in the CNS during MS and many other CNS pathologies.

### Acid-Sensing Ion Channels in Astrocytes

Astrocytes fulfill a variety of roles in the CNS, such as regulating the formation and maintenance of the BBB (Abbott et al., 2006). After brain injury and disease, astrocytes can become reactive, participating in repair cascades and gene upregulation, and forming an astroglial scar (Zamanian et al., 2012; Anderson et al., 2016). Reactive astrocytes are key players in CNS disease, an area reviewed recently by Dossi et al. (2018). Stimulation of astrocytes by cytokines such as interferon gamma (IFN−γ) leads to expression of the antigen-presenting major histocompatibility complex II (MHC II) on the cell surface alongside other costimulatory molecules such as intercellular adhesion molecule 1 (ICAM-1) (Wong et al., 1984; Lee et al., 2000). MHC II expression occurs in MS plaques and may contribute to the inflammatory cascade through antigen presentation to T cells (Zeinstra et al., 2000). Ponath et al. (2018) recently reviewed the differing roles of astrocytes in MS.

ASIC1, ASIC2a, ASIC3 are expressed in the nucleus of rat astrocytes, and astrocytes express an ASIC-like current that is blocked by amiloride (Huang et al., 2010; Yu et al., 2015). Yang et al. (2016) noted increased ASIC1a expression in the membrane and cytoplasm of reactive astrocytes from the hippocampi of deceased TLE patients, findings which were replicated in a mouse model of chronic epileptogenesis. To explore normal astrocytic function, the group used primary cultures from wild-type mice. At 24 h after stimulation with LPS, astrocytes upregulated ASIC1a and exhibited increased Ca^2+^ influx upon exposure to pH 6.0, which was significantly reduced by PcTx1.

Yang et al. (2016) showed that seizure frequency in TLE mice is reduced by knockdown of astrocytic ASIC1a, with restoration of the *ASIC1* gene increasing the frequency of spontaneous seizures. Thus, in contrast to what is believed regarding epilepsy and the beneficial function of ASIC1a (likely via neuronal expression), these data suggest that activation of astrocytic ASIC1a causes pathological Ca^2+^ influx and contributes to the pathogenesis of TLE.

### Acid-Sensing Ion Channels in Oligodendrocytes

Oligodendrocytes deliver critical trophic support to neurons, producing the proteins and lipids that comprise the myelin sheath that wrap around and insulate axons, and they engage in significant cross-talk with microglia during the process of myelination (Peferoen et al., 2014). Oligodendrocytes secrete cytokines such as C-C motif chemokine ligand 2 (CCL-2) and IL-8 in response to neuroborreliosis, the neurological manifestation of Lyme’s disease (Ramesh et al., 2012). IL-8 and CCL-2 are chemotaxic for a number of peripheral immune cells, such as neutrophils and T cells respectively (Turner et al., 2014).

Oligodendrocytes are especially sensitive to ischemic insults (Bradl and Lassmann, 2010), and resident ASICs may contribute to ischemia-induced injury of these cells. ASIC1 is upregulated in oligodendrocytes in chronic brain lesions of patients with MS, and use of amiloride in patients provided neuroprotection at primary stages of the disease (Arun et al., 2013). ASIC1a, ASIC2a/b (non-selective primers were used, and thus ASIC2a and ASIC2b could not be distinguished), and ASIC4 mRNA were found in oligodendrocytes, and ASIC-specific inward currents recorded from oligodendrocyte lineage cells (OLCs) were inhibited by PcTx1 (Feldman et al., 2008). Cultured mouse oligodendrocytes express ASIC1 (ASIC1a and ASIC1b were not separable) and they were protected from acidosis-induced damage by PcTx1, as were oligodendrocytes derived from *ASIC1* KO mice (Vergo et al., 2011). Thus, the combined data suggest that ASIC1 activation contributes to oligodendrocyte injury during and after periods of tissue acidosis.

### Acid-Sensing Ion Channels in Macrophages

Macrophages are key players in inflammation and autoimmune disease. They produce NO under acidic conditions and promote demyelination in the CNS (Bellocq et al., 1998; Yamasaki et al., 2014; Li and Barres, 2018). RT-PCR and Western blots were used to demonstrate expression of ASIC1 and ASIC3 in the cytoplasm of macrophages (Kong et al., 2013). When exposed to acidic conditions (pH 6.5), bone marrow-derived macrophages (BMMs) increase their rate of phagocytosis (Kong et al., 2013). Extracellular acidosis also causes an upregulation of key cell surface markers related to BMM maturation (e.g., CD80, CD86 and MHC II). These effects were blocked by application of amiloride before exposure to tissue acidosis. Interestingly, expression of IL-10—thought to be a beneficial anti-inflammatory cytokine—was increased in macrophages after acid exposure.

Thus, the limited available data suggest that ASICs can modulate the macrophage response to acidic microenvironments. CNS macrophages/monocytes are believed to be a primary contributor to MS demyelination, and ASICs may contribute to their response in this pathology (Yamasaki et al., 2014).

### Acid-Sensing Ion Channels in T Cells

T cells play a major role in coordinating and executing multiple functions of the adaptive immune response. Autoreactive T cells that have lost their ability to differentiate self (i.e., host cells and antigens) from non-self (i.e., foreign/pathogenic cells and antigens) are major players in autoimmune diseases such as MS. In the first study that identified a role for ASIC1a in the neuropathology of MS, ASIC1b, ASIC3 and ASIC4 were identified at the mRNA level in mouse T cells, with the presence of ASIC1 protein confirmed in these immune cells via Western blot (Friese et al., 2007). In this study, no obvious function for ASIC1 in T cells was observed based on responses of wild-type and ASIC1 knockout T cells in terms of proliferation and cytokine secretion. Thus, the role of ASICs in T cells remains to be determined, but they do not seem to play a role in these cells in the context of MS, at least not in the mouse EAE model.

### Acid-Sensing Ion Channels in Dendritic Cells

Mature DCs are professional antigen presenting cells that constitute a key part of the inflammatory cascade, forming a major link between the innate and adaptive immune systems. The expression of MHC II and co-stimulatory molecules such as CD86 on their cell surface allows them to present antigens to T cells leading to T cell activation and proliferation (Reis e Sousa, 2006; Dudek et al., 2013).

When mature DCs are exposed to acidic conditions (pH 6.5) for a moderate time period (∼4 h), the expression of their specific cell-surface markers is increased (Vermeulen et al., 2004). Human DCs exposed to acidosis increase production of proinflammatory IL-12 and the authors suggest that this triggers a bias toward a proinflammatory Th1 response (Martínez et al., 2007). The acidosis-induced increase in cell-surface marker expression was replicated using cultured DCs derived from mouse bone marrow, a response that was blocked by amiloride (Tong et al., 2011). Using non-specific antibodies for ASIC subtypes (i.e., not distinguishing between splice isoforms), Tong et al. (2011) found that mouse DCs express ASIC protein in the cytoplasm (ASIC1 and ASIC2), on the plasma membrane (ASIC2), in the endoplasmic reticulum and perinuclear regions (ASIC1), and in the mitochondria (ASIC3). Functional surface expression of ASICs was confirmed using patch-clamp electrophysiology experiments and the observation of amiloride-sensitive, acidosis-induced inward currents with a pH_50_ of ∼6.0. Acidosis (pH 6.5) increased the antigen-presenting ability of DCs as assessed by increased ability to stimulate T cell proliferation, and this effect was blocked by amiloride (Tong et al., 2011).

These results strongly suggest that ASIC activation on DCs enhances expression of cell-surface proteins involved in antigen presentation and subsequent T cell activation and proliferation. As T cells can be highly destructive to healthy tissue in chronic inflammatory conditions, acidosis-enhanced communication between these two CNS-invading peripheral immune cells may exacerbate CNS damage in disease (Korn and Kallies, 2017).

### Acid-Sensing Ion Channels in Natural Killer Cells

Data from the human binary protein interactome (Luck et al., 2020) indicate an interaction between ASIC1a and the killer cell immunoglobulin-like receptor 3DL3, a key regulator of NK cell function. Reduced levels of NK cells have also been implicated in depression (in which immune responses tend to be impaired). Brain acidity is altered in several mental health conditions (Coryell et al., 2009; Suzuki et al., 2017), but it is not yet clear whether this impacts on NK levels or function or whether ASICs have any role in these changes.

## Neuropathologies in Which Acid-Sensing Ion Channels Are Implicated

ASIC1a is expressed in the cell body, dendritic arbor and postsynaptic dendritic spines of brain neurons (Wemmie et al., 2002; Alvarez de La Rosa et al., 2003; Vukicevic and Kellenberger, 2004; Zha et al., 2006). The brain can suffer significant pH reductions during CNS pathologies, falling to as low as 6.0 during severe cerebral ischemia (Rehncrona, 1985). Given that the pH_50_ for activation of ASIC1a in human cortical neurons is 6.6 (Li et al., 2010), such drops in brain pH are sufficient to robustly activate ASIC1a. ASIC1a expression is upregulated in both dorsal root ganglia (DRG) and spinal dorsal horn neurons in response to inflammation, and in DRGs this upregulation is suppressed by ASIC inhibitors (Voilley et al., 2001; Duan et al., 2007). Prevention of ASIC1a endocytosis caused elevated death of cortical neurons exposed to acidosis in wild-type, but not ASIC1a knockout, mice (Zeng et al., 2013).

### Multiple Sclerosis and Other Neurodegenerative Diseases

Axonal degeneration plays a major role in MS, with associated inflammation causing dysfunctional activity of mitochondria (Waxman, 2006). Using the EAE model of MS, it was found that mice in which the *ASIC1* gene was genetically inactivated or the channel was inhibited with amiloride exhibited marked axonal preservation (Friese et al., 2007). Consistent with the hypothesis that ASIC1a is activated during MS, increased levels of lactate are found in brain lesions of MS patients (Bitsch et al., 1999). ASICs are also expressed in microglia, astrocytes and oligodendrocytes, major players in MS that engage in crosstalk (Domingues et al., 2016). While microglia are the primary phagocytotic cells in the CNS, astrocytes also possess the ability to phagocytose neuronal debris, axonal mitochondria, and pathological protein aggregates (Lee and Chung, 2021). In aging mice, microglia appear to accumulate myelin debris at the same time as myelin degeneration (Hill et al., 2018). Indeed, Popovich et al. (2002) demonstrated that direct activation of “CNS macrophages” (covering both invading peripheral macrophages and CNS resident microglia) results in axonal damage and demyelination. This conclusion has gained strong support over the past 20 years and activated microglia in particular seem to represent promising targets in MS (Geladaris et al., 2021). As described above, ASICs are functionally expressed in both macrophages and microglia, and contribute to their activation during periods of acidosis. Thus, the protection afforded by ASIC1a inhibition in the EAE mouse model of MS is possibly due to decreased macrophage/microglial activation as well as direct neuroprotection.

In a PD model, knockout of ASIC1a made no difference to the number of dopaminergic neurons, the key subset of neurons that are lost in this pathology and lead to motor impairment (Komnig et al., 2016). A significant build-up of lactate has been observed across all brain regions in animal models of HD, and in the frontal cortex of HD patients (Harms et al., 1997; Dautry et al., 1999; Tsang et al., 2006). Administration of an amiloride derivative in a HD model caused a reduction in polyQ aggregation, one potential effector in this pathology, both *in vivo* and *in vitro* (Wong et al., 2008). Although the authors state that this indicates a role for ASIC1a in HD, their use of a non-specific ASIC inhibitor means that one cannot rule out the potential involvement of other channels/transporters in this neurodegenerative disease.

### Seizures

Epileptic seizures are associated with increased acidity in the brain, and upon CO_2_ administration the resultant hypercapnia allows for seizure termination through lowering of brain pH (Lennox, 1928; Wang and Sonnenschein, 1955; Miller, 2011). Overexpression of ASIC1a in mice appears to limit the duration and progression of chemoconvulsant-induced seizures, but not the number, and knockout of the gene has the converse effect (Ziemann et al., 2008). Furthermore, the beneficial effect of CO_2_ inhalation requires ASIC1a to interrupt induced seizures. This may be due to activation of ASICs causing generation of action potentials in inhibitory interneurons, although ASICs are also expressed in excitatory pyramidal neurons (Cho and Askwith, 2008; Ziemann et al., 2008; Weng et al., 2010). Another study found that amiloride suppressed pilocarpine-induced seizures, but the use of this pan-ASIC inhibitor makes it difficult to relate this outcome to specific involvement of ASIC1a (Liang et al., 2015). Consistent with ASIC1a playing a role in seizure termination, hippocampal levels of ASIC1a are elevated in patients with temporal lobe epilepsy (TLE) and in epileptic mice (Yang et al., 2016). It has been suggested that in a neurotypical system, seizures should be self-limiting due to the ASIC1a response to increased acidity at seizure onset; in support of this hypothesis, a single nucleotide polymorphism (SNP) in the *ASIC1* gene is associated with TLE (Lv et al., 2011; Wemmie et al., 2013).

### Mental Health

Patients with schizophrenia exhibit lower pH and increased lactate in the cerebellum (Halim et al., 2008), whereas patients with bipolar disorder show decreased pH in the dorsolateral prefrontal cortex and increased lactate in gray matter regions of the brain (Dager et al., 2004; Sun et al., 2006). The frontal cortex and brain homogenate of rodent models replicated these findings with both increased lactate and reduced pH observed (Halim et al., 2008; Hagihara et al., 2017). ASIC1a is abundantly expressed in the amygdala, a brain region that has been described as a chemosensor that promotes fear behavior after detection of CO_2_ and/or acidosis (Wemmie et al., 2003; Coryell et al., 2007; Ziemann et al., 2009). Activation or inhibition/disruption of ASIC1a and altered expression of the *ASIC1* gene all modify fear-related behaviors. Coryell et al. (2007) found that loss of the *ASIC1* gene affected neuronal activity in the amygdala after exposure to fear-inducing odors. Not only do *ASIC1* KO mice exhibit reduced fear, overexpression of the channel increases fear responses (Wemmie et al., 2002, 2004). In humans, two SNPs in the *ASIC1* gene are associated with anxiety, linking with amygdala structure and function and also risk of panic disorder (Smoller et al., 2014). Lastly, Coryell and colleagues used a mouse model to examine the link between ASIC1a and depression. They found that pharmacological inhibition and or genetic ablation of ASIC1a reduced depression like-symptoms, while restoring the gene to the amygdala returned responses back to baseline (Coryell et al., 2009).

### Central Nervous System Trauma and Ischemia

As described earlier, vascular disruption due to trauma can result in acidification of the brain, and the resultant drop in pH is often sufficient to activate neuronal ASIC1a (Xiong et al., 2004; Li et al., 2010; Chassagnon et al., 2017). It was originally envisaged that neuronal ASIC1a might exacerbate ischemia-induced brain injury by contributing to excitotoxicity by virtue of its ability to mediate flux of Ca^2+^ into neurons in addition to Na^+^ (Xiong et al., 2004; Yermolaieva et al., 2004). However, recent studies suggests that, at least in the brain, activation of ASIC1a also leads to recruitment and activation of receptor-interacting serine/threonine-protein kinase 1 (RIPK1), a key mediator of necroptosis (Wang et al., 2015, 2020). Remarkably, this activation of RIPK1 occurs independently of ion flow through the channel (Wang et al., 2015).

How does ASIC1a contribute to ischemic injury when it is subject to rapid steady-state desensitisation (SSD) when exposed to sustained acidic pH *in vitro* (Gründer and Chen, 2010), and is distinguished from other ASICs by a reduced responsiveness to successive acid stimulations, a phenomenon known as tachyphylaxis (Chen and Grunder, 2007)? One might assume that these properties would limit the persistence of ASIC1a currents during sustained periods of tissue acidosis *in vivo*. However, a variety of ensuing biochemical events act to persistently activate ASIC1a during a sustained drop in tissue pH. First, ASIC1a currents are potentiated by several ischemia-related factors, including: (i) extracellular lactate which increases from a basal level of 1–2 mM to 12–20 mM during ischemia (Allen and Attwell, 2002; Gonzalez-Inchauspe et al., 2020); (ii) membrane stretch resulting from the rise in extracellular [K^+^] (Allen and Attwell, 2002); and (iii) CaMKII phosphorylation of ASIC1a (Gao et al., 2005). Second, during ischemic stroke, the increase in extracellular spermine prolongs ASIC1a currents and enhances recovery from SSD (Duan et al., 2011). Third, the increase in intracellular Ca^2+^ inhibits tachyphylaxis. Fourth, arachidonic acid, which is elevated during stroke due to activation of phospholipase A_2_ (PLA_2_), potentiates ASIC1a currents and enhances the sustained component of the current (Allen and Attwell, 2002). Finally, and perhaps most importantly, the train of cell-death signaling set off by the initial activation of ASIC1a continues regardless of the subsequent state of the channel. This includes activation of necroptosis (Wang et al., 2015; Redd et al., 2021), apoptosis (Song et al., 2019), and the stimulation of cell executioners such as calcium-activated proteases, endonucleases, and PLA_2_ due to the increased levels of intracellular Ca^2+^ (Yermolaieva et al., 2004; Xiong et al., 2007).

Thus, despite its susceptibility to desensitization (which has largely been studied under control conditions in *in vitro* assays), activation of ASIC1a during sustained tissue acidosis is a major contributor to ischemic injury, as evidenced by the fact that genetic ablation or specific pharmacological inhibition of ASIC1a greatly reduces the tissue damage caused by ischemic stroke (Xiong et al., 2004; McCarthy et al., 2015; Chassagnon et al., 2017). Genetic knockout or knockdown of ASIC1a, as well as pharmacological inhibition of the channel, also provide neuroprotective effects post-SCI (Hu et al., 2011; Koehn et al., 2016). However, it should be noted that these studies have focused exclusively on the role of ASIC1a in CNS neurons. Despite the critical role of immune cells in damage progression after CNS trauma, very few studies have explored whether the resident population of ASIC1a, or other ASICs, in immune cells contributes to ischemic jury of the CNS.

## Conclusion

**FIGURE 5:**
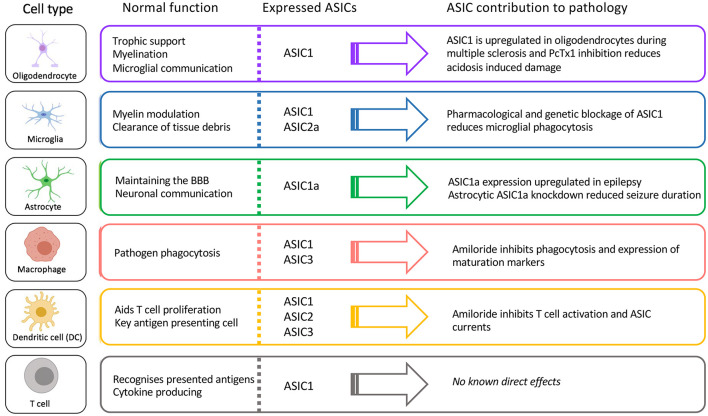
Function, ASIC profile, and putative role of ASICs in CNS immune cells.

Here we have described what is currently known about the expression and role of ASICs in immune cells known to contribute to a wide range of neuropathologies. We highlight the fact that ASICs are expressed in many key CNS-resident and infiltrating immune cells involved in these pathologies and are not only expressed in neurons (Figure 5). Thus, it is important to consider whether the therapeutic benefit provided by ASIC inhibitors in some of these pathologies is exerted not only via effects on neurons, but also on immune cells.

A common theme across immune cells is alterations to phagocytosis after exposure to acidity. Ineffective phagocytosis contributes to autoimmune diseases such as systemic lupus erythematosus (SLE) and polyarthritis (Nagata et al., 2010). Whether ASICs are the primary mediators of acidity-induced alterations in phagocytosis remains to be determined. Their role may depend on the type and severity of the pathology, in turn leading to beneficial or negative outcomes, as seen for other receptors such as P2X7 (Savio et al., 2018). The CNS is heterogenous in its way of dealing with acidity (Chesler, 2003) and immune cells also respond differently to the “type” of acidity (Kellum et al., 2004). The resultant complexity of pH-induced effects results in a multitude of unique outcomes dependent on CNS region and immune cell type, as well as the ways in which acidity is induced. To unravel the role of immune-cell ASICs in neuropathology will likely require standardization of models, including the method of acidosis induction.

Another caveat of current research on the role of ASICs in immune-cell function is the widespread use of amiloride as a broad-spectrum ASIC inhibitor. Unfortunately, amiloride has targets outside of the ASIC family, including sodium-hydrogen antiporter 1 (NHE-1), which is involved in acid-sensing and pH regulation by immune cells. Amiloride can also inhibit TRPP3 channels (Dai et al., 2007), which are thought to be involved in acid sensing in mouse spinal cord and alkaline sensing in lamprey (Huang et al., 2006; Dai et al., 2007; Jalalvand et al., 2016). This raises the question of whether the beneficial effects of amiloride in human MS patients is solely due to inhibition of ASICs (Arun et al., 2013). Much more potent and specific ASIC inhibitors are available, such as Hi1a (Chassagnon et al., 2017) and PcTx1 (Escoubas et al., 2000) for ASIC1a, mambalgins for rodent ASIC1a and ASIC1b (Diochot et al., 2012) (although they potentiate human ASIC1b under the levels of acidosis likely encountered *in vivo;* Cristofori-Armstrong et al., 2021) and the sea-anemone peptide APETx2 for ASIC3 (Diochot et al., 2004), and they should be used in preference to amiloride wherever possible. Unfortunately, there are no specific inhibitors available for ASIC1b and ASIC2a. A final concern with investigations of ASIC protein expression is ongoing challenges with the specificity of available ASIC antibodies. As highlighted by Lin et al. (2015), examples of lack of specificity include “positive” ASIC1a staining in a pan-ASIC1a knockout mouse (Vig et al., 2014). Thus, due to the limitations of currently available commercial antibodies, antibody staining data should always be combined with proteomics and/or functional data using subtype-specific inhibitors to provide confirmation of ASIC localization.

There are some studies where subtype-specific inhibitors or specific genetic ablation has been used to definitively demonstrate a role for ASICs in CNS pathologies, such as ischemic stroke (Pignataro et al., 2007; McCarthy et al., 2015; Chassagnon et al., 2017). However, even in these instances, the relative contribution of neuronal and immune-cell ASICs was not considered. Our understanding of how immune-cell ASICs contribute to neuropathology would be significantly enhanced by utilizing technologies that allow direct study of these channels in specific immune cells. This was achieved in an eloquent study by Yang et al. (2016) through knockdown and then restoration of ASIC1a solely on astrocytes in a mouse model of TLE. Such techniques, particularly in combination with selective pharmacological tools, will allow determination of whether pan-inhibition of specific ASIC subtypes or their inhibition only in specific cell types is likely to be therapeutically useful.

In conclusion, the role of ASIC in immune cells is an exciting frontier in neurological research. Merging CNS immune function studies with cutting-edge molecular techniques will provide greater insight into whether immune-cell ASICs are likely to be useful drug targets for CNS disorders.

## Author Contributions

VF conceived the study and wrote the first draft of the manuscript. LR, MR, and GK revised and streamlined the manuscript. MR and GK contributed funding and mentored VF. All authors contributed to the article and approved the submitted version.

## Conflict of Interest

The authors declare that the research was conducted in the absence of any commercial or financial relationships that could be construed as a potential conflict of interest.

## Publisher’s Note

All claims expressed in this article are solely those of the authors and do not necessarily represent those of their affiliated organizations, or those of the publisher, the editors and the reviewers. Any product that may be evaluated in this article, or claim that may be made by its manufacturer, is not guaranteed or endorsed by the publisher.

## Abbreviations

AD: Alzheimer’s disease
ASIC: acid-sensing ion channel
BBB: blood–brain barrier
BCSFB: blood-cerebrospinal fluid barrier
BMM: bone marrow-derived macrophage
BSCB: blood-spinal cord barrier
CCL-2: C-C motif chemokine ligand 2
CNS: central nervous system
CRAC: calcium release-activated calcium channel
CXCL-8: interleukin-8
DAMP: damage-associated molecular pattern
DC: dendritic cell
Deg: degenerin
DRG: dorsal root ganglion
DS: lactate-based dialysis solution
EAE: experimental autoimmune encephalomyelitis
ENaC: epithelial sodium channel
HD: Huntington’s disease
ICAM-1: intercellular adhesion molecule 1
i.c.v.: intracerebroventricular
IFN−γ: interferon gamma
IL: interleukin
LPS: lipopolysaccharide
MCT: monocarboxylate transporter
MHC: major histocompatibility complex
MPO: myeloperoxidase
MS: multiple sclerosis
NHE-1: sodium-hydrogen antiporter 1
NK: natural killer
NO: nitric oxide
NOS: nitric oxide synthase
OLC: oligodendrocyte lineage cell
PECAM-1: platelet endothelial cell adhesion molecule 1
PAMP: pathogen associated molecular pattern
PD: Parkinson’s disease
PNS: peripheral nervous system
PRR: pattern recognition receptor
RAW 264.7: mouse macrophage-like cell line
RIPK1: receptor-interacting serine/threonine-protein kinase 1
ROS: reactive oxygen species
SCI: spinal cord injury
SLE: systemic lupus erythematosus
SNP: single nucleotide polymorphism
TBI: traumatic brain injury
TCR: T cell receptor
TLE: temporal lobe epilepsy
TLR: Toll-like receptor
TNF: tumor necrosis factor
TRP: transient receptor potential.
