# Supplementary material for: Acid-Sensing Ion Channels: Expression and Function in Resident and Infiltrating Immune Cells in the Central Nervous System
Source: Front Cell Neurosci. 2021 Sep 17;15:738043. doi: 10.3389/fncel.2021.738043 (PMC8484650; doi:10.3389/fncel.2021.738043)
Supplement: Supplementary file 1 [file Data_Sheet_1.PDF]

SUPPLEMENTARY

Supplementary Table 1: Tissue expression, physiology and pathology of ASICs in mammals. Additional information on expression can be found on the Human Protein Atlas website at www.proteinatalas.org.

Isoform	Tissue distribution	Physiology and pathology
ASIC1a	**General:** Glioblastoma cell line (Tian et al. 2017). Vascular smooth muscle (Chung et al. 2010; Jernigan 2015). Retina (Ettaiche et al. 2006). Bone (Jahr et al. 2005; Swain et al. 2012). Lung (Nitta et al. 2014). Tongue (Richter et al. 2004). Bladder (Kobayashi et al. 2009). Gut (Dong et al. 2011). Oligodendrocyte-lineage cells (Feldman et al. 2008). Heart (Redd et al. 2021). Kidney (Song et al. 2019).<br><br>**PNS:** Dorsal root ganglion, sciatic nerve (Alvarez de La Rosa et al. 2003). Cochlear spiral ganglion (González-Garrido et al. 2015).<br><br>**CNS:** Spinal cord (Alvarez de La Rosa et al. 2003; Wu et al. 2004; Duan et al. 2007; Baron et al. 2008). Brain: Forebrain, midbrain, brainstem, pons, cerebellum, hippocampus, cortex (Waldmann et al. 1997; Wemmie et al. 2002; Cho and Askwith 2008).<br><br>Oligodendrocyte-lineage cells (Feldman et al. 2008).	Retinal function (Ettaiche et al. 2006).<br>Seizure duration (Ziemann et al. 2008).<br>Fear conditioning (Wemmie et al. 2002; Wemmie et al. 2004; Coryell et al. 2007; Ziemann et al. 2009).<br>Anxiety (Coryell et al. 2007; Smoller et al. 2014).<br>Multiple sclerosis (Friese et al. 2007).<br>Depression (Coryell et al. 2009).<br>Synaptic plasticity (Zha et al. 2006).<br>Addiction behaviour (Kreple et al. 2014).<br>Lung hypertension, proliferation and migration of lung cancer cells (Nitta et al. 2014; Wu et al. 2017)<br>Upregulation in joint capsule of frozen shoulder (Cho et al. 2015).<br>Contribute to migration of cells in pancreatic cancer (with ASIC3) (Zhu et al. 2017).<br>Upregulated by inflammation/inflammatory pain in spinal cord (Wu et al. 2004; Duan et al. 2007).<br>Secondary damage after stroke and spinal cord injury (Xiong et al. 2004; Xiong et al. 2006; Hu et al. 2011; Koehn et al. 2016; Chassagnon et al. 2017).<br>Ischemic injury after reperfusion in heart and kidney (Song et al. 2019; Redd et al. 2021).
ASIC1b	**General:** Inner ear (Bässler et al. 2001). Cochlear hair cells (Ugawa et al. 2006) .<br>**PNS:** DRG (Chen et al. 1998).<br>**CNS:** Spinal cord and brain (in rat): Forebrain, midbrain, brainstem, pons, cerebellum, hippocampus (Alvarez de La Rosa et al. 2003).	Possible role in nociception (Diochot et al. 2012; Verkest et al. 2018; Chang et al. 2019).
ASIC2a	**General:** Retina (Ettaiche et al. 2004). Heart (Abboud and Benson 2015). Taste cells (Ugawa 2003). Bone (Jahr et al. 2005). Glioma cells (Vila-Carriles et al. 2006). Bladder (Corrow et al. 2010)<br>**PNS:** Dorsal root ganglion (Price et al. 1996; García-Añoveros et al. 2001; Alvarez de la Rosa et al. 2002; Cabo et al. 2015). Sciatic nerve (Alvarez de la Rosa et al. 2002). Cochlear spiral ganglion (Peng et al. 2004; González-Garrido et al. 2015). Skin (Montaño et al. 2009; Cabo et al. 2015).	Mechanosensation (García-Añoveros et al. 2001; Montaño et al. 2009; Cabo et al. 2015).<br>Visual transduction (Ettaiche et al. 2004).<br>Invasion and metastases in colorectal cancer (Zhou et al. 2017).<br>Noise susceptibility (Peng et al. 2004).<br>Taste (Ugawa 2003).<br>Upregulation in joint capsule of frozen shoulder (Cho et al. 2015).

	**CNS:** Spinal cord (Price et al. 1996; Wu et al. 2004; Baron et al. 2008). Brain: amygdala, hippocampus, cerebellum, olfactory bulb, cortex and brain stem (Price et al. 1996). Oligodendrocyte-lineage cells (Feldman et al. 2008).	Upregulated by inflammation in the spinal cord (Wu et al. 2004).<br>Cardiovascular homeostasis (Abboud and Benson 2015).<br>Upregulation after cystitis (Corrow et al. 2010).
ASIC2b	**General:** Retina (Ettaiche et al. 2004). Taste cells (Ugawa 2003).<br>**PNS:** Dorsal root ganglion (Price et al. 1996; Alvarez de la Rosa et al. 2002). Sciatic nerve (Alvarez de la Rosa et al. 2002). Cochlear spiral ganglion (González-Garrido et al. 2015).<br>**CNS:** Spinal cord (Wu et al., 2004; Baron et al., 2008). Brain: amygdala, hippocampus, cerebellum, olfactory bulb, cortex and brain stem (Price et al. 1996).	Visual transduction (Ettaiche et al. 2004).<br>Cardiovascular homeostasis (Abboud and Benson 2015).<br>Taste (Ugawa 2003).
ASIC3	**General:** Glioblastoma cell line (Tian et al. 2017). Muscle arterioles (Molliver et al. 2005). Muscle (Walder et al. 2011). Joints (Ikeuchi et al. 2009). Gut (Yiangou et al. 2001). Heart (Sutherland et al. 2001; Abboud and Benson 2015). Inner ear (Hildebrand et al. 2004). Retina and cochlea (Maubaret et al. 2002). Bone (Jahr et al. 2005; Tong et al. 2011). Testis (Ishibashi and Marumo 1998). Intestine, adipose and bladder (Yiangou et al. 2001; Jones et al. 2005; Kobayashi et al. 2009; Corrow et al. 2010). Skin (Price et al. 2001).<br>**PNS:** DRG/sensory neurons (Price et al. 2001; Molliver et al. 2005; Deval et al. 2008). Sciatic nerve (Alvarez de la Rosa et al. 2002). Colon afferent neuronal fibers (Jones et al. 2005). Cochlear spiral ganglion (González-Garrido et al. 2015).<br>**CNS**: Spinal cord (Delaunay et al. 2012).<br>**CNS (low expression):** Brain (increased in epilepsy) (Cao et al. 2016).	Visceral mechanosensitivity and inflammation (Yiangou et al. 2001; Jones et al. 2005).<br>Metaboreception (Molliver et al. 2005).<br>Cutaneous touch and nociception (Price et al. 2001; Walder et al. 2011).<br>Inflammatory pain (Deval et al. 2008; Ikeuchi et al. 2009).<br>Itch sensation (Peng et al. 2015).<br>Cardiovascular homeostasis (Abboud and Benson 2015).<br>Hearing (Hildebrand et al. 2004).<br>Contribute to migration of cells in pancreatic cancer (with ASIC1) (Zhu et al. 2017).<br>Inhibition of epilepsy (Cao et al. 2016).<br>Role in angina (Sutherland et al. 2001; Yagi et al. 2006).<br>Hyperalgesia in muscle (Walder et al. 2011).<br>Upregulation in joint capsule of frozen shoulder (Cho et al. 2015).<br>Upregulation after cystitis (Corrow et al. 2010).
ASIC4	**General:** Inner ear, pituitary gland (Gründer et al. 2000).<br>**PNS** (low): DRG (neonate) and sympathetic ganglion (neonate) (Lin et al. 2015). Sciatic nerve (Alvarez de la Rosa et al. 2002).<br>**CNS**: spinal cord, brain stem, olfactory bulb, pituitary gland, striatum, hippocampus, amygdala, thalamus, hypothalamus, cerebral cortex, cerebellum (Gründer et al. 2000; Lin et al. 2015; Hoshikawa et al. 2017). Oligodendrocyte-lineage cells (Feldman et al. 2008).	Role in fear suppression (Lin et al. 2015).

References

Abboud, F. M., and Benson, C. J. (2015) ASICs and cardiovascular homeostasis. *Neuropharmacology* 94, 87–98. doi: 10.1016/j.neuropharm.2014.12.017.

Alvarez de La Rosa, D., Krueger, S. R., Kolar, A., Shao, D., Fitzsimonds, R. M., and Canessa, C. M. (2003) Distribution, subcellular localization and ontogeny of ASIC1 in the mammalian central nervous system. *J. Physiol.* 546, 77–87. doi:

Alvarez de la Rosa, D., Zhang, P., Shao, D., White, F., and Canessa, C. M. (2002) Functional implications of the localization and activity of acid-sensitive channels in rat peripheral nervous system. *Proc. Natl. Acad. Sci. USA* 99, 2326–2331. doi: 10.1073/pnas.042688199.

Baron, A., Voilley, N., Lazdunski, M., and Linguеglia, E. (2008) Acid sensing ion channels in dorsal spinal cord neurons. *J. Neurosci.* 28, 1498–1508. doi: 10.1523/jneurosci.4975-07.2008.

Bässler, E. L., Ngo-Anh, T. J., Geisler, H. S., Ruppersberg, J. P., and Gründer, S. (2001) Molecular and functional characterization of acid-sensing ion channel (ASIC) 1b. *J. Biol. Chem.* 276, 33782–33787. doi: 10.1074/jbc.M104030200.

Cabo, R., Alonso, P., Viña, E., Vázquez, G., Gago, A., Feito, J., et al. (2015) ASIC2 is present in human mechanosensory neurons of the dorsal root ganglia and in mechanoreceptors of the glabrous skin. *Histochem. Cell Biol.* 143, 267–276. doi: 10.1007/s00418-014-1278-y.

Cao, Q., Wang, W., Gu, J., Jiang, G., Wang, K., Xu, Z., et al. (2016) Elevated expression of acid-sensing ion channel 3 inhibits epilepsy via activation of interneurons. *Mol. Neurobiol.* 53, 485–498. doi: 10.1007/s12035-014-9014-0.

Chang, C.-T., Fong, S. W., Lee, C.-H., Chuang, Y.-C., Lin, S.-H., and Chen, C.-C. (2019) Involvement of acid-sensing ion channel 1b in the development of acid-induced chronic muscle pain. *Front. Neurosci.* 13, 1247. doi: 10.3389/fnins.2019.01247.

Chassagnon, I. R., McCarthy, C. A., Chin, Y. K., Pineda, S. S., Keramidas, A., Mobli, M., et al. (2017) Potent neuroprotection after stroke afforded by a double-knot spider-venom peptide that inhibits acid-sensing ion channel 1a. *Proc. Natl. Acad. Sci. USA* 114, 3750–3755. doi: 10.1073/pnas.1614728114.

Chen, C. C., England, S., Akopian, A. N., and Wood, J. N. (1998) A sensory neuron-specific, proton-gated ion channel. *Proc. Natl. Acad. Sci. USA* 95, 10240–10245. doi: 10.1073/pnas.95.17.10240.

Cho, C. H., Lho, Y. M., Ha, E., Hwang, I., Song, K. S., Min, B. W., et al. (2015) Up-regulation of acid-sensing ion channels in the capsule of the joint in frozen shoulder. *Bone Joint J.* 97-B, 824–829. doi: 10.1302/0301-620x.97b6.35254.

Cho, J.-H., and Askwith, C. (2008) Presynaptic release probability is increased in hippocampal neurons from ASIC1 knockout mice. *J. Neurophysiol.* 99, 426–441. doi:

Chung, W.-S., Farley, J. M., Swenson, A., Barnard, J. M., Hamilton, G., Chiposi, R., et al. (2010) Extracellular acidosis activates ASIC-like channels in freshly isolated cerebral artery smooth muscle cells. *Am. J. Physiol. – Cell Physiol.* 298, C1198–C1208. doi: 10.1152/ajpcell.00511.2009.

Corrow, K., Girard, B. M., and Vizzard, M. A. (2010) Expression and response of acid-sensing ion channels in urinary bladder to cyclophosphamide-induced cystitis. *Am. J. Physiol. – Renal Physiol.* 298, F1130–F1139. doi: 10.1152/ajprenal.00618.2009.

Coryell, M. W., Wunsch, A. M., Haenfler, J. M., Allen, J. E., Schnizler, M., Ziemann, A. E., et al. (2009) Acid-sensing ion channel-1a in the amygdala, a novel therapeutic target in depression-related behavior. *J. Neurosci* 29, 5381–5388. doi: 10.1523/jneurosci.0360-09.2009.

Coryell, M. W., Ziemann, A. E., Westmoreland, P. J., Haenfler, J. M., Kurjakovic, Z., Zha, X.-M., et al. (2007) Targeting ASIC1a reduces innate fear and alters neuronal activity in the fear circuit. *Biol. Psychiatry* 62, 1140–1148. doi: 10.1016/j.biopsych.2007.05.008.

Delaunay, A., Gasull, X., Salinas, M., Noël, J., Friend, V., Lingueglia, E., et al. (2012) Human ASIC3 channel dynamically adapts its activity to sense the extracellular pH in both acidic and alkaline directions. *Proc. Natl. Acad. Sci. USA* 109, 13124–13129. doi: 10.1073/pnas.1120350109.

Deval, E., Noël, J., Lay, N., Alloui, A., Diochot, S., Friend, V., et al. (2008) ASIC3, a sensor of acidic and primary inflammatory pain. *EMBO J.* 27, 3047–3055. doi: 10.1038/emboj.2008.213.

Diochot, S., Baron, A., Salinas, M., Douguet, D., Scarzello, S., Dabert-Gay, A. S., et al. (2012) Black mamba venom peptides target acid-sensing ion channels to abolish pain. *Nature* 490, 552–555. doi: 10.1038/nature11494.

Dong, X., Ko, K. H., Chow, J., Tuo, B., Barrett, K. E., and Dong, H. (2011) Expression of acid-sensing ion channels in intestinal epithelial cells and their role in the regulation of duodenal mucosal bicarbonate secretion. *Acta Physiologica* 201, 97–107. doi: 10.1111/j.1748-1716.2010.02207.x.

Duan, B., Wu, L. J., Yu, Y. Q., Ding, Y., Jing, L., Xu, L., et al. (2007) Upregulation of acid-sensing ion channel ASIC1a in spinal dorsal horn neurons contributes to inflammatory pain hypersensitivity. *J. Neurosci.* 27, 11139–11148. doi: 10.1523/JNEUROSCI.3364-07.2007.

Ettaiche, M., Deval, E., Cougnon, M., Lazdunski, M., and Voilley, N. (2006) Silencing acid-sensing ion channel 1a alters cone-mediated retinal function. *J. Neurosci.* 26, 5800–5809. doi: 10.1523/JNEUROSCI.0344-06.2006.

Ettaiche, M., Guy, N., Hofman, P., Lazdunski, M., and Waldmann, R. (2004) Acid-sensing ion channel 2 is important for retinal function and protects against light-induced retinal degeneration. *J. Neurosci.* 24, 1005–1012. doi: 10.1523/JNEUROSCI.4698-03.2004.

Feldman, D., Horiuchi, M., Keachie, K., McCauley, E., Bannerman, P., Itoh, A., et al. (2008) Characterization of acid-sensing ion channel expression in oligodendrocyte-lineage cells. *Glia* 56, 1238–1249. doi: 10.1002/glia.20693.

Friese, M. A., Craner, M. J., Etzensperger, R., Vergo, S., Wemmie, J. A., Welsh, M. J., et al. (2007) Acid-sensing ion channel-1 contributes to axonal degeneration in autoimmune inflammation of the central nervous system. *Nat. Med.* 13, 1483–1489. doi: 10.1038/nm1668.

García-Añoveros, J., Samad, T. A., Žuvela-Jelaska, L., Woolf, C. J., and Corey, D. P. (2001) Transport and localization of the DEG/ENaC ion channel BNaC1α to peripheral mechanosensory terminals of dorsal root ganglia neurons. *J. Neurosci.* 21, 2678–2686. doi: 10.1523/JNEUROSCI.21-08-02678.2001.

González-Garrido, A., Vega, R., Mercado, F., López, I. A., and Soto, E. (2015) Acid-sensing ion channels expression, identity and role in the excitability of the cochlear afferent neurons. *Front. Cell. Neurosci.* 9, 483–483. doi: 10.3389/fncel.2015.00483.

Gründer, S., Geisler, H., Bässler, L., and Ruppersberg, J. P. (2000) Cloning of ASIC 4, a new member of acid-sensing ion channels from pituitary gland. *Eur. J. Neurosci.* 12, 22. doi:

Hildebrand, M. S., de Silva, M. G., Klockars, T., Rose, E., Price, M., Smith, R. J. H., et al. (2004) Characterisation of DRASIC in the mouse inner ear. *Hearing Res.* 190, 149–160. doi: 10.1016/s0378-5955(04)00015-2.

Hoshikawa, M., Kato, A., Hojo, H., Shibata, Y., Kumamoto, N., Watanabe, M., et al. (2017) Distribution of ASIC4 transcripts in the adult wild-type mouse brain. *Neuroscience Lett.* 651, 57–64. doi: https://doi.org/10.1016/j.neulet.2017.03.054.

Hu, R., Duan, B., Wang, D. S., Yu, Y., Li, W. G., Luo, H. S., et al. (2011) Role of acid-sensing ion channel 1a in the secondary damage of traumatic spinal cord injury. *Ann. Surg.* 254, 353–362. doi: 10.1097/SLA.0b013e31822645b4.

Ikeuchi, M., Kolker, S. J., and Sluka, K. A. (2009) Acid-sensing ion channel 3 expression in mouse knee joint afferents and effects of carrageenan-induced arthritis. *The Journal of Pain* 10, 336–342. doi: https://doi.org/10.1016/j.jpain.2008.10.010.

Ishibashi, K., and Marumo, F. (1998) Molecular cloning of a DEG/ENaC sodium channel cDNA from human testis. *Biochem. Biophys. Res. Commun.* 245, 589–593. doi: https://doi.org/10.1006/bbrc.1998.8483.

Jahr, H., van Driel, M., van Osch, G. J. V. M., Weinans, H., and van Leeuwen, J. P. T. M. (2005) Identification of acid-sensing ion channels in bone. *Biochem. Biophys. Res. Commun.* 337, 349–354. doi:

Jernigan, N. L. (2015) Smooth muscle acid-sensing ion channel 1: pathophysiological implication in hypoxic pulmonary hypertension. *Exp. Physiol.* 100, 111–120. doi: 10.1113/expphysiol.2014.081612.

Jones, R. C., 3rd, Xu, L., and Gebhart, G. F. (2005) The mechanosensitivity of mouse colon afferent fibers and their sensitization by inflammatory mediators require transient receptor potential vanilloid 1 and acid-sensing ion channel 3. *J. Neurosci.* 25, 10981–10989. doi: 10.1523/jneurosci.0703-05.2005.

Kobayashi, H., Yoshiyama, M., Zakoji, H., Takeda, M., and Araki, I. (2009) Sex differences in the expression profile of acid-sensing ion channels in the mouse urinary bladder: a possible involvement in irritative bladder symptoms. *BJU Int.* 104, 1746–1751. doi: 10.1111/j.1464-410X.2009.08658.x.

Koehn, L. M., Noor, N. M., Dong, Q., Er, S.-Y., Rash, L. D., King, G. F., et al. (2016) Selective inhibition of ASIC1a confers functional and morphological neuroprotection following traumatic spinal cord injury. *F1000Research* 5, 1822. doi: 10.12688/f1000research.9094.2.

Kreple, C., Lu, Y., Taugher, R., Schwager-Gutman, A., Du, J., Stump, M., et al. (2014) Acid-sensing ion channels contribute to synaptic transmission and inhibit cocaine-evoked plasticity. *Nat. Neurosci.* 17, 1083–1091. doi: 10.1038/nn.3750.

Lin, S.-H., Chien, Y.-C., Chiang, W.-W., Liu, Y.-Z., Lien, C.-C., and Chen, C.-C. (2015) Genetic mapping of ASIC4 and contrasting phenotype to ASIC1a in modulating innate fear and anxiety. *Eur. J. Neurosci.* 41, 1553–1568. doi: 10.1111/ejn.12905.

Maubaret, C., Delettre, C., Sola, S., and Hamel, C. P. (2002) Identification of preferentially expressed mRNAs in retina and cochlea. *DNA Cell Biol.* 21, 781–791. doi: 10.1089/104454902320908432.

Molliver, D. C., Immke, D. C., Fierro, L., Paré, M., Rice, F. L., and McCleskey, E. W. (2005) ASIC3, an acid-sensing ion channel, is expressed in metaboreceptive sensory neurons. *Mol. Pain* 1, 35–35. doi: 10.1186/1744-8069-1-35.

Montaño, J. A., Calavia, M. G., García-Suárez, O., Suarez-Quintanilla, J. A., Gálvez, A., Pérez-Piñera, P., et al. (2009) The expression of ENa+C and ASIC2 proteins in Pacinian corpuscles is differently regulated by TrkB and its ligands BDNF and NT-4. *Neurosci. Lett.* 463, 114–118. doi: https://doi.org/10.1016/j.neulet.2009.07.073.

Nitta, C. H., Osmond, D. A., Herbert, L. M., Beasley, B. F., Resta, T. C., Walker, B. R., et al. (2014) Role of ASIC1 in the development of chronic hypoxia-induced pulmonary hypertension. *Am J. Physiol. – Heart Circ. Physiol.* 306, H41–H52. doi: 10.1152/ajpheart.00269.2013.

Peng, B.-G., Ahmad, S., Chen, S., Chen, P., Price, M. P., and Lin, X. (2004) Acid-sensing ion channel 2 contributes a major component to acid-evoked excitatory responses in spiral ganglion neurons and plays a role in noise susceptibility of mice. *J. Neurosci.* 24, 10167–10175. doi: 10.1523/JNEUROSCI.3196-04.2004.

Peng, Z., Li, W.-G., Huang, C., Jiang, Y.-M., Wang, X., Zhu, Michael X., et al. (2015) ASIC3 mediates itch sensation in response to coincident stimulation by acid and nonproton ligand. *Cell Rep.* 13, 387–398. doi: https://doi.org/10.1016/j.celrep.2015.09.002.

Price, M. P., McIlwrath, S. L., Xie, J., Cheng, C., Qiao, J., Tarr, D. E., et al. (2001) The DRASIC cation channel contributes to the detection of cutaneous touch and acid stimuli in mice. *Neuron* 32, 1071–1083. doi: https://doi.org/10.1016/S0896-6273(01)00547-5.

Price, M. P., Snyder, P. M., and Welsh, M. J. (1996) Cloning and expression of a novel human brain Na^+ channel. *J. Biol. Chem.* 271, 7879–7882. doi: 10.1074/jbc.271.14.7879.

Redd, M. A., Scheuer, S. E., Saez, N. J., Yoshikawa, Y., Chiu, H. S., Gao, L., et al. (2021) Therapeutic inhibition of acid sensing ion channel 1a recovers heart function after ischemia-reperfusion injury. *Circulation*, in press. doi: 10.1161/CIRCULATIONAHA.121.054360.

Richter, T. A., Dvoryanchikov, G. A., Roper, S. D., and Chaudhari, N. (2004) Acid-sensing ion channel-2 is not necessary for sour taste in mice. *J. Neurosci.* 24, 4088–4091. doi: 10.1523/JNEUROSCI.0653-04.2004.

Smoller, J. W., Gallagher, P. J., Duncan, L. E., McGrath, L. M., Haddad, S. A., Holmes, A. J., et al. (2014) The human ortholog of acid-sensing ion channel gene ASIC1a is associated with panic disorder and amygdala structure and function. *Biol. Psychiat.* 76, 902–910. doi: 10.1016/j.biopsych.2013.12.018.

Song, N., Lu, Z., Zhang, J., Shi, Y., Ning, Y., Chen, J., et al. (2019) Acid-sensing ion channel 1a is involved in ischaemia/reperfusion induced kidney injury by increasing renal epithelia cell apoptosis. *J. Cell. Mol. Med.* 23, 3429–3440. doi: 10.1111/jcmm.14238.

Sutherland, S. P., Benson, C. J., Adelman, J. P., and McCleskey, E. W. (2001) Acid-sensing ion channel 3 matches the acid-gated current in cardiac ischemia-sensing neurons. *Proc. Natl. Acad. Sci. USA* 98, 711–716. doi: 10.1073/pnas.98.2.711.

Swain, S. M., Parameswaran, S., Sahu, G., Verma, R. S., and Bera, A. K. (2012) Proton-gated ion channels in mouse bone marrow stromal cells. *Stem Cell Res.* 9, 59–68. doi: 10.1016/j.scr.2012.04.005.

Tian, Y., Bresenitz, P., Reska, A., El Moussaoui, L., Beier, C. P., and Gründer, S. (2017) Glioblastoma cancer stem cell lines express functional acid sensing ion channels ASIC1a and ASIC3. *Scientific Reports* 7. doi: 10.1038/s41598-017-13666-9.

Tong, J., Wu, W.-N., Kong, X., Wu, P.-F., Tian, L., Du, W., et al. (2011) Acid-sensing ion channels contribute to the effect of acidosis on the function of dendritic cells. *J. Immunol.* 186, 3686–3692. doi:

Ugawa, S. (2003) Identification of sour-taste receptor genes. *Anatom. Sci. Int.* 78, 205–210. doi: 10.1046/j.0022-7722.2003.00062.x.

Ugawa, S., Inagaki, A., Yamamura, H., Ueda, T., Ishida, Y., Kajita, K., et al. (2006) Acid-sensing ion channel-1b in the stereocilia of mammalian cochlear hair cells. *NeuroReport* 17, 1235–1239. doi: 10.1097/01.wnr.0000233093.67289.66.

Verkest, C., Piquet, E., Diochot, S., Dauvois, M., Lanteri-Minet, M., Lingueglia, E., et al. (2018) Effects of systemic inhibitors of acid-sensing ion channels 1 (ASIC1) against acute and chronic mechanical allodynia in a rodent model of migraine. *Br. J. Pharmacol.* 175, 4154–4166. doi: 10.1111/bph.14462.

Vila-Carriles, W. H., Kovacs, G. G., Jovov, B., Zhou, Z. H., Pahwa, A. K., Colby, G., et al. (2006) Surface expression of ASIC2 inhibits the amiloride-sensitive current and migration of glioma cells. *J. Biol. Chem.* 281, 19220–19232. doi: 10.1074/jbc.M603100200.

Walder, R. Y., Gautam, M., Wilson, S. P., Benson, C. J., and Sluka, K. A. (2011) Selective targeting of ASIC3 using artificial miRNAs inhibits primary and secondary hyperalgesia after muscle inflammation. *Pain* 152, 2348–56. doi: 10.1016/j.pain.2011.06.027.

Waldmann, R., Champigny, G., Bassilana, F., Heurteaux, C., and Lazdunski, M. (1997) A proton-gated cation channel involved in acid-sensing. *Nature* 386, 173. doi: 10.1038/386173a0.

Wemmie, J. A., Chen, J., Askwith, C. C., Hruska-Hageman, A. M., Price, M. P., Nolan, B. C., et al. (2002) The acid-activated ion channel ASIC contributes to synaptic plasticity, learning, and memory. *Neuron* 34, 463–477. doi: 10.1016/s0896-6273(02)00661-x.

Wemmie, J. A., Coryell, M. W., Askwith, C. C., Lamani, E., Leonard, A. S., Sigmund, C. D., et al. (2004) Overexpression of acid-sensing ion channel 1a in transgenic mice increases acquired fear-related behavior. *Proc. Natl. Acad. Sci. USA* 101, 3621–3626. doi: 10.1073/pnas.0308753101.

Wu, L. J., Duan, B., Mei, Y. D., Gao, J., Chen, J. G., Zhuo, M., et al. (2004) Characterization of acid-sensing ion channels in dorsal horn neurons of rat spinal cord. *J. Biol. Chem.* 279, 43716–43724. doi: 10.1074/jbc.M403557200.

Wu, Y., Gao, B., Xiong, Q. J., Wang, Y. C., Huang, D. K., and Wu, W. N. (2017) Acid-sensing ion channels contribute to the effect of extracellular acidosis on proliferation and migration of A549 cells. *Tumour Biol.* 39, 1010428317705750. doi: 10.1177/1010428317705750.

Xiong, Z.-G., Zhu, X.-M., Chu, X.-P., Minami, M., Hey, J., Wei, W.-L., et al. (2004) Neuroprotection in ischemia: blocking calcium-permeable acid-sensing ion channels. *Cell* 118, 687–698. doi:

Xiong, Z. G., Chu, X. P., and Simon, R. P. (2006) Ca^{2+}-permeable acid-sensing ion channels and ischemic brain injury. *J. Membr. Biol.* 209, 59–68. doi: 10.1007/s00232-005-0840-x.

Yagi, J., Wenk Heather, N., Naves Ligia, A., and McCleskey Edwin, W. (2006) Sustained currents through asic3 ion channels at the modest pH changes that occur during myocardial ischemia. *Circ. Res.* 99, 501–509. doi: 10.1161/01.RES.0000238388.79295.4c.

Yiangou, Y., Facer, P., Smith, J. A., Sangameswaran, L., Eglen, R., Birch, R., et al. (2001) Increased acid-sensing ion channel ASIC-3 in inflamed human intestine. *Eur. J. Gastroenterol. Hepatol.* 13, 891–896. doi: 10.1097/00042737-200108000-00003.

Zha, X. M., Wemmie, J. A., Green, S. H., and Welsh, M. J. (2006) Acid-sensing ion channel 1a is a postsynaptic proton receptor that affects the density of dendritic spines. *Proc. Natl. Acad. Sci. USA* 103, 16556–16561. doi: 10.1073/pnas.0608018103.

Zhou, Z.-H., Song, J.-W., Li, W., Liu, X., Cao, L., Wan, L.-M., et al. (2017) The acid-sensing ion channel, ASIC2, promotes invasion and metastasis of colorectal cancer under acidosis by activating the calcineurin/NFAT1 axis. *J./ Exp. Clin. Cancer Res.* 36, 130. doi: 10.1186/s13046-017-0599-9.

Zhu, S., Zhou, H. Y., Deng, S. C., Deng, S. J., He, C., Li, X., et al. (2017) ASIC1 and ASIC3 contribute to acidity-induced EMT of pancreatic cancer through activating Ca^{2+}/RhoA pathway. *Cell Death Dis.* 8, e2806. doi: 10.1038/cddis.2017.189.

Ziemann, A. E., Allen, J. E., Dahdaleh, N. S., Drebot, II, Coryell, M. W., Wunsch, A. M., et al. (2009) The amygdala is a chemosensor that detects carbon dioxide and acidosis to elicit fear behavior. *Cell* 139, 1012–1021. doi: 10.1016/j.cell.2009.10.029.

Ziemann, A. E., Schnizler, M. K., Albert, G. W., Severson, M. A., Howard, M. A., Welsh, M. J., et al. (2008) Seizure termination by acidosis depends on ASIC1a. *Nat. Neurosci.* 11, 816–822. doi: 10.1038/nn.2132.
